# ECG-image-database: large-scale paired ECG images and time-series with real-world artifacts; a foundation for computerized ECG digitization and analysis

**DOI:** 10.1088/1361-6579/ae85b2

**Published:** 2026-07-27

**Authors:** Matthew A Reyna, Deepanshi, Audrey T Weigle, Zuzana Koscova, Kiersten Campbell, Kshama Kodthalu Shivashankara, Soheil Saghafi, Sepideh Nikookar, Mohsen Motie-Shirazi, Yashar Kiarashi, Salman Seyedi, Mohammadsina Hassannia, Agnar M Bjørnstad, Elias Stenhede, Arian Ranjbar, Gari D Clifford, Reza Sameni

**Affiliations:** 1 Department of Biomedical Informatics, Emory University, Atlanta, GA 30322, United States of America; 2 School of Electrical and Computer Engineering, Georgia Institute of Technology, Atlanta, GA 30332, United States of America; 3 Medical Technology & E-health, Akershus University Hospital, 1478 Lørenskog, Norway; 4 Faculty of Medicine, University of Oslo, 0316 Oslo, Norway; 5 Department of Biomedical Engineering, Georgia Institute of Technology, Atlanta, GA 30332, United States of America

**Keywords:** electrocardiogram, ECG image, image digitization, ECG diagnosis, public database

## Abstract

*Objective.* We present electrocardiogram (ECG)-Image-Database, comprising ECG image datasets paired with the underlying time-series signals. The database is designed to reflect real-world digitization challenges by incorporating diverse scanning, imaging, and physical artifacts commonly encountered in clinical and archival ECG records. It currently includes ECG images of records from Emory Healthcare (Atlanta, USA), the PTB-XL dataset from Physikalisch-Technische Bundesanstalt (Berlin, Germany), and Akershus University Hospital (Lørenskog, Norway). *Approach.* ECG images from Emory Healthcare and PTB-XL were generated using ECG-Image-Kit, an open-source toolkit that converts 12-lead ECG time series into realistic printout-style images. The toolkit introduces controlled digital distortions, including noise, wrinkles, stains, and perspective changes. A total of 1977 ECG records (977 PTB-XL and 1000 Emory Healthcare) were processed to create high-fidelity ECG printouts. These images were further subjected to physical degradation—such as soaking, staining, and mold exposure—before being scanned or photographed under varying lighting and capture conditions. In addition, we captured mobile photographs of the original ECG images displayed on computer monitors. This process resulted in 35 595 images. Independently, ECG images from Akershus University Hospital were captured from thermal-paper ECG printouts from 266 patients acquired during routine clinical care. Each printout underwent natural handling degradation and was repeatedly scanned and photographed, yielding 1596 images. *Main results.* ECG-Image-Database contains 37 191 images derived from 2243 ECG records across three countries. The dataset spans clean scans, noisy photographs, and severe physical deterioration, and includes heterogeneous ECG layouts and acquisition conditions. Each record is linked to its corresponding digital ECG time series, enabling supervised training and benchmarking of digitization and classification methods. Subsets of this resource were used in the PhysioNet Challenge 2024 and a subsequent 2025–2026 Kaggle challenge on ECG image digitization and classification. The database is structured for scalability and future expansion. *Significance.* By providing paired ECG images and signals across diverse real-world artifact conditions, ECG-Image-Database supports robust development of methods for recovering ECG time series from images and for direct image-based interpretation, helping preserve diagnostic value from non-digital ECG archives.

## 1. Introduction

The electrocardiogram (ECG) remains the most accessible tool for cardiovascular disease (CVD) assessment, with over three million ECGs generated daily by clinicians (Shenasa *et al* 2015), and millions more each day from wearable and personal devices. It is estimated that there are billions of digital diagnostic ECGs globally (Tison *et al* 2019), with a comparable number in hardcopy formats, including microfilm, paper, and scanned images (Davis *et al* 1973). Paper ECGs are gradually being replaced by digital ECGs—although much more slowly in low-income and low-resource regions—but legacy ECG records contain invaluable information on individual history, rare events, and the evolution of CVDs across generations and geographic regions. Moreover, the proprietary nature of many ECG acquisition systems means that sharing raw ECG data is often difficult or costly, and printing the ECG as a PDF, image, or on physical paper is frequently the only option for moving the data beyond commercial walled gardens. Due to natural deterioration and limited funding for physical archives, decades of non-digital ECG archives worldwide will soon be destroyed before we can learn from them and leverage them for training machine learning (ML) models for algorithmic diagnosis of CVDs. This loss would be irreversible and an almost incalculable setback for cardiovascular research, as the ECG is the only biological signal that has been recorded for over a century without significant changes in its acquisition protocol. Over recent decades, most countries have experienced substantial shifts in cardiovascular health, and longitudinal analysis of paper records has the potential to map these changes in an unprecedented manner. Moreover, the populations from which we may learn the most, and which are least represented in commercial products, are also the most likely to have paper ECGs.

Modern ECG machines and wearable technologies enable the collection of massive digital ECG datasets. However, it will take decades for digital archives to replace the lost diagnostic and demographic information contained in physical archives, especially in low-resource areas and low- and middle-income countries (LMICs), where electronic health records are rare, leading to the loss of demography-specific clinical data. Even in high-income regions, printed ECGs remain common and are routinely circulated between clinicians as hardcopies or pictures for adjudication and training purposes. Recently, specialized public forums have formed on social media platforms , with hundreds of thousands of expert members who regularly share de-identified (i.e. protected health information (PHI) has been redacted) ECG images and discuss diagnoses and clinical outcomes, serving as a modern form of traditional *grand rounds* or *case studies* in medical education and inpatient care.

To preserve printed ECGs, some research teams and healthcare systems in affluent regions scan and archive them in image formats. However, there are insufficient incentives to dedicate the time and effort required to preserve most printed ECGs. Scanning alone is not enough to ensure the usefulness of an ECG because: (i) ECG images are incompatible with most computerized ECG annotation software, which is typically trained on and designed to analyze ECG time series; (ii) ECG images are not currently searchable automatically for annotations and anomalies; (iii) existing technologies for ECG diagnosis are not available at the point of care; and (iv) it is difficult to determine *a priori* whether scanned ECGs are of sufficient quality for algorithmic ECG diagnosis. To address these issues, research teams have developed image processing pipelines, supervised computer vision software, and, more recently, deep learning-based methods for ECG digitization. The highlights of these contributions are reviewed in section 2.

The ECG digitization process can be understood as a sequence of steps: (i) scanning or photographing physical ECGs as images; (ii) detecting and extracting ECG time-series data from the images and mapping pixels into physical ECG units (see appendix A); and (iii) annotating or labeling the ECG data. The later steps of this process can inform the earlier ones; for example, a device could automatically rescan a paper ECG when the image is not clear enough for time series extraction. However, to date, paper-based ECG digitization and algorithmic ECG diagnosis have not been integrated using ECG domain knowledge. Therefore, the added value of learning from historical ECG images remains largely unrealized.

While the availability of ECG time-series datasets is increasing, there remains a significant gap in standardized datasets that pair these time series with corresponding ECG images across a wide range of image qualities. Currently, no standard dataset provides underlying ECG time-series data alongside ECG images spanning high-quality scans, photographs, low-quality grayscale or black-and-white formats, or images affected by environmental artifacts such as imaging and scanning issues, natural deterioration, and other common distortions.

To address this critical gap, we developed and publicly released ECG-Image-Database (Reyna *et al* 2026), which we introduce in this paper, consisting of paired ECG images and time-series records. The database is version-controlled and will continue to expand over time. Currently, ECG-Image-Database consists of ECG printouts from standard, widely accessible datasets with real-world imaging artifacts, including ECGs from the PTB-XL dataset, Germany (Wagner *et al* 2020, 2022); Emory Healthcare, USA (Reyna *et al* 2021); and Akershus University Hospital, Norway. These images were printed, subjected to natural artifacts, and compiled into a very large dataset encompassing a wide spectrum of imaging and scanning distortions. This comprehensive dataset enables the development and training of more robust and generalizable digitization models, helping ensure that these tools can effectively handle the diverse conditions encountered in real-world clinical ECG images.

Importantly, although evident in the signal and image processing communities, there is a significant difference between ECG data in time-series format and images of ECG signals. Throughout this paper, ‘ECG time series’ refers to the digital samples of an ECG, i.e. a sequence of ECG signal amplitude values over time, which is the preferred format for ECG analysis and annotation software; ‘non-digital ECG’ refers to ECGs in a physical hardcopy format (such as paper or microfilm) that have not been digitized; ‘scanned ECG’ or ‘ECG image’ refers to images of ECGs obtained either through photography or scanning of non-digital ECGs, or stored directly in an image format by an ECG machine at the point of care. Although the latter is considered a digital representation of the ECG, the corresponding ECG time series are generally not directly available (unless stored as a ‘vector PDF’ with the raw ECG data embedded within it as lines and curves, in which case the ECG can be obtained via programmatic extraction from vector PDFs, which is a separate software task beyond the scope of this work). Throughout this article, ‘ECG digitization’ refers to the process of retrieving ECG time-series data from images, and ‘ECG annotation’ refers to the process of assigning diagnostic labels to an ECG (in image or time-series formats), either by a human or a machine/algorithm.

## 2. Background and significance

To provide context, we review major highlights from prior research on ECG image digitization and clinical measurements, which motivate the development of standardized ECG image datasets.

Traditional image processing and computer vision pipelines focus on digitizing ECG data by removing background grids and noise to extract clean signals. However, these approaches differ substantially in their techniques and algorithms. Common methods include image processing techniques such as binary morphological image operations (Tun 2017), grayscale thresholding (Ravichandran *et al* 2013), and linear filtering (Ganesh *et al* 2021). Additionally, several studies have used optical character recognition (OCR) to capture patient demographic information and integrate it into medical records (Silva *et al* 2008, Ravichandran *et al* 2013, Ganesh *et al* 2021). Some methods have also explored cost-effective, non-hardware solutions (Silva *et al* 2008, Virgin and Baskar 2018).

Fortune *et al* (2022) developed an algorithm to capture ECG morphology and beat timing with high precision, validated through statistical measures. Zhang *et al* (1987) applied image processing techniques such as histogram filtering to remove noise, enabling efficient archival storage. Widman and Hines (1991) employed optical scanners and demonstrated improved signal fidelity through adjustments in paper speed and amplifier gain. Reproducible methods for scanning and analyzing QT intervals were introduced in Bhullar *et al* (1993) and Wang and Mital (1996), enhancing the clinical utility of ECG digitization techniques. Lobodzinski *et al* (2002) developed an optical ECG waveform recognition method to digitize paper ECGs using statistical filtering and image processing. This work was further extended in Lobodzinski *et al* (2003), where an XML-based format was proposed for storing digitized ECG data. Badilini *et al* (2005) introduced ECGScan, employing active contour modeling to detect and extract ECG waveforms from paper records. Mitra *et al* (2004) applied Fourier transform techniques to convert ECG images into digital signals, focusing on frequency analysis. Karsikas *et al* (2007) proposed a digitization method involving the scanning of paper ECGs followed by image processing to correct misalignment and remove grid noise while preserving ECG signal integrity.

More recently, advanced ML pipelines have been proposed for converting ECG images into time-series data. As demonstrated by Holkeri *et al* (2018), traditional ML techniques can be leveraged to refine and improve the accuracy of clinical measurements derived from ECG images. Baydoun *et al* (2019) applied neural networks to convert ECG scans into actionable data.

However, these approaches have largely been constrained by the availability of small, manufacturer-specific datasets, limiting their broader applicability and generalizability. Despite these advances, the full potential of deep learning models such as ResNet (He *et al* 2016) and large-scale datasets such as ImageNet (Deng *et al* 2009), which have significantly advanced image processing applications in other domains, remained largely unexplored in ECG digitization until recently. The adoption of these more powerful models could enable more robust and scalable solutions for ECG image processing, particularly by addressing the limitations of small datasets and manufacturer-specific variations. However, generic computer vision tools have yet to address the unique challenges of ECG digitization (Waits and Soliman 2017), including the diversity of diagnostic ECG devices ranging from single- to twelve-lead systems, variations in ECG paper standards, creases, wrinkles, non-uniform fading of ink, and other forms of physical wear, as well as the extraction of computerized or handwritten text such as patient identifiers, lead names, and notes from low-resolution images or scans. Waits and Soliman (2017) categorized the challenges of digitization approaches (prior to the last decade) into several key issues: grid-related problems, time efficiency, alignment difficulties, signal quality and noise concerns, and validation challenges, particularly validation on large and diverse samples. More recent approaches based on deep learning have the potential to learn ECG-specific features directly from images without requiring extraction of the underlying ECG time-series data (Brisk *et al* 2019), which used a deep neural network to classify down-sampled images generated from the raw training signals of the PhysioNet Challenge 2017 (Clifford *et al* 2017) dataset. However, the quality metrics of generic computer vision deep models differ from those required for ECG diagnosis, and the volume of available ECG images with simultaneous ECG time-series data and clinical annotations remains inadequate for training large deep learning models. Data augmentation using synthetic ECG images (time-series data printed on paper ECG grids) may enable algorithms to diagnose ECG images directly. More recently, Shivashankara *et al* (2024) introduced deep neural networks to enhance the ECG digitization process, demonstrating promising results in handling variability in ECG images across devices using data augmentation with synthetic paper ECG images. Similarly, Demolder *et al* (2025) introduced a two-stage, deep-learning-based approach for ECG digitization and classification across a variety of image types, sources, and variations. This method has since been implemented in PMcardio, a commercially available technology for ECG image digitization.

Most recently, major breakthroughs were achieved through the George B. Moody PhysioNet Challenge 2024 on ECG image digitization and classification (Reyna *et al* 2024), and the 2025–2026 Kaggle challenge on ECG image digitization (Reyna *et al* 2025). In both challenges, models were trained on a public portion of ECG-Image-Database and evaluated on hidden validation and test subsets. In this work, we provide a complete description of the construction of ECG-Image-Database and publicly release the data.

## 3. Generating realistic ECG images from time-series data

ECG-Image-Kit is an open-source toolbox developed to generate synthetic ECG images that closely replicate real-world ECG printouts (Deepanshi *et al* 2024, Shivashankara *et al* 2024). The primary purpose of this toolkit is to support the training and development of data-intensive deep learning models for ECG digitization, addressing the critical need to convert legacy, non-digital ECG archives into digital formats compatible with modern diagnostic AI/ML tools. This tool was used to create the initial electronic version of ECG-Image-Database presented in this work.

The toolkit generates realistic ECG images from time-series data by simulating ECGs printed on thermal paper or by inkjet and laser printers. It applies configurable distortions that reflect real-world digitization challenges, including overlaid printed and handwritten text artifacts such as lead labels, calibration pulses, patient information, and diagnostic notes, which often overlap the ECG waveform. ECG-Image-Kit also simulates physical paper artifacts, including wrinkles and creases, using image processing techniques such as image quilting and blurring. In addition, the toolkit applies perspective distortions, imaging artifacts, and noise (Gaussian, Poisson, and salt-and-pepper) to model camera angle variations and scanning imperfections. Color temperature adjustments are further used to simulate aging and environmental effects on ECG thermal paper.

ECG-Image-Kit also provides flexibility in customizing the format of ECG leads displayed in synthetic images, supporting both standard and non-standard configurations. This feature is particularly important for replicating the diverse range of ECG formats encountered in clinical practice across different regions of the world. For example, standard paper ECGs typically display all 12 leads in 2.5 s segments across four rows, sweeping from left to right in time. Additionally, leads II, V1, V2, and/or V5 are often shown as continuous 10 s strips at the bottom for rhythm analysis. Older ECG machines recorded the 2.5 s segments for different leads asynchronously, meaning that the segments did not correspond to the same time frame. This is an important consideration for ECG digitization algorithms, as they cannot rely on synchrony between channel segments to improve the extracted ECG time series through multichannel post-processing. However, the longer strips do overlap with the shorter segments. The default mode of ECG-Image-Kit generates the most common 12-lead format (3 rows by 4 columns of 2.5 s segments, with a long 10 s strip at the bottom of the page). This default behavior can be modified through optional parameters detailed in (Deepanshi *et al* 2024). Additionally, the toolkit enables the generation of large batches of ECG images with random, yet controlled, levels of artifacts and noise, making it well suited for creating the very large datasets required for training deep learning models.

ECG-Image-Kit has been implemented in Python. It provides command-line flags that allow users to control various aspects of generating ECG images from ECG time-series data stored in WFDB format (Moody *et al* 2022). For example, users can adjust image resolution (with the -r flag), add handwritten text distortions (using the –hw_tex| flag), or introduce paper-like wrinkles and creases (with the –wrinkles flag). The toolkit can process either individual ECG records or entire directories of ECG data. By specifying the input path and desired output directory, users can generate ECG images either in batch mode or for individual records. This flexibility allows granular control over the number and characteristics of generated images, making it possible to create diverse ECG representations with varying levels of artifacts and distortions.

ECG-Image-Kit was used in the George B. Moody PhysioNet Challenge 2024: Digitization and Classification of ECG Images to generate realistic ECG images with natural artifacts (Reyna *et al* 2024 ), and in the 2025–2026 Kaggle challenge: PhysioNet Digitization of ECG Images (Reyna *et al* 2025).

## 4. ECG data sources

ECG-Image-Database includes three time-series datasets with clinical annotations, creating a rich collection of ECG images containing diverse printing, scanning, and imaging artifacts while preserving access to the ground-truth ECG time series. These datasets are described below.

### 4.1. The PTB-XL dataset

The PTB-XL dataset, provided publicly by the Physikalisch-Technische Bundesanstalt institute (Berlin, Germany), consists of 21 799 clinical 12-lead ECG records, each 10 s long, from 18 869 patients (Wagner *et al* 2020). These data were collected using Schiller AG devices between October 1989 and June 1996. The patient group is 52% male and 48% female, with ages ranging from 0 to 95 years (median age: 62). The dataset includes a broad range of heart conditions and healthy control samples, categorized into five main diagnostic classes: Normal ECG (9514 records), Myocardial Infarction (5469), ST/T Change (5235), Conduction Disturbance (4898), and Hypertrophy (2649). The ECG data are stored in WFDB format at 500 Hz, with downsampled versions at 100 Hz provided for convenience. Metadata for each record are provided in a CSV file and include identifiers, demographic details, ECG diagnostics, and signal quality information. Additional fields track annotations such as heart axis, noise, and artifacts. The dataset includes a recommended 10-fold train-test split, with records in folds 9 and 10 having undergone human validation for label quality. PTB-XL+ (Strodthoff *et al* 2023) is a supplementary dataset that provides additional ECG features and algorithmic annotations from the PTB-XL dataset using three software tools: (i) the University of Glasgow ECG Analysis Program version R30.4.2 (Macfarlane *et al* 2005); (ii) GE Healthcare’s Marquette™ 12SL™ (GE Healthcare 2019); and (iii) the open-source tool ECGDeli version 1.1 (Pilia *et al* 2021). These annotations include median beats, fiducial points, and automatic diagnostic statements, allowing users to train and evaluate ML models with enhanced ECG metadata.

We used rejection sampling to select a representative subset of 977 ECGs from the PTB-XL dataset for creating the presented ECG images. These ECGs approximately preserve the univariate distributions of the patient attributes and classes from the source dataset.

### 4.2. The Emory Healthcare dataset

We used a random subset of 1000 ECG records from the Emory Healthcare dataset, which features a diverse patient population in Georgia, USA. Access to this dataset was approved by Emory University’s Institutional Review Board under the PhysioCrowd protocol STUDY-00 007 353. This dataset is a subset of the Harvard–Emory ECG Database (HEEDB) (Koscova *et al* 2026). It consists of 12-lead clinical ECGs recorded between 2010 and 2022 by GE ECG machines of different generations from healthcare subjects of diverse demographic backgrounds. The data were originally stored in XML format and included ECG-based measurements and algorithmic annotations generated by GE’s software. Due to the extended data collection period, we re-annotated the ECG time-series data using GE Healthcare’s latest Marquette™ 12SL™ software, resulting in unified relabeling of all selected records.

### 4.3. The Akershus University Hospital dataset

Akershus University Hospital (Ahus) in Lørenskog, Norway, serves more than 10% of the Norwegian population and covers a wide sociodemographic range, from Oslo suburbs with greater socioeconomic challenges to more rural communities. At Ahus, 12-lead ECGs are routinely printed on paper to support bedside interpretation and clinical review, while the corresponding digital time series are automatically archived in the hospital’s Picture Archiving and Communication System (PACS) for long-term storage. This workflow provides a natural opportunity for paired paper–digital data collection. Between February and April 2025, we prospectively collected 12-lead paper ECGs at the Department of Cardiology. Staff were instructed to archive ECG papers instead of discarding them at patient discharge, ensuring that the paper ECGs underwent routine clinical handling, including paper bending and occasional handwritten annotations such as weight, height, or blood pressure.

The corresponding digital ECGs were recorded on SCHILLER AT-102 G2 or AT-180 systems and stored with a duration of 10 s at a sampling rate of 1000 Hz, with the first 5 s overlapping with the paper ECG. On-board preprocessing was applied, consisting of a band-pass filter from 0.51 to 42.25 Hz and a powerline notch filter centered at 50 Hz. The digital signals were stored in the hospital’s PACS in accordance with the DICOM standard. All paper ECGs were printed on SCHILLER thermal recording paper #2.157 050 in a two-page Cabrera format with six leads per page, recorded at 50 mm s$ ^{-1}$ and 10 mm mV$ ^{-1}$. Both pages display the same 5 s of the recorded ECG.

Paper ECGs were matched to their digital counterparts in PACS using national identification numbers and acquisition timestamps. In cases where multiple ECGs existed for the same patient on the same day, manual inspection was performed. When only a date of birth was available, the PACS was queried using this information, and candidate matches were manually verified.

The Ahus dataset was used for validation of an ECG digitization algorithm reported in Stenhede *et al* (2026). The subset released here comprises 266 ECGs from 266 unique patients. Of these, 99 were female, 153 were male, and 14 had unknown sex due to missing national identification numbers and no manually entered sex. Among the 210 patients with valid national identification numbers, 109 (52%) had heart failure, 94 (45%) had atrial fibrillation or flutter, 93 (44%) had chronic ischemic heart disease, 75 (36%) had essential hypertension, and 58 (28%) had other cardiac arrhythmias. Most ECGs were collected in the Department of Cardiology; some were recorded in other departments and later transferred as part of the patient’s hospital stay.

**Figure 1.**
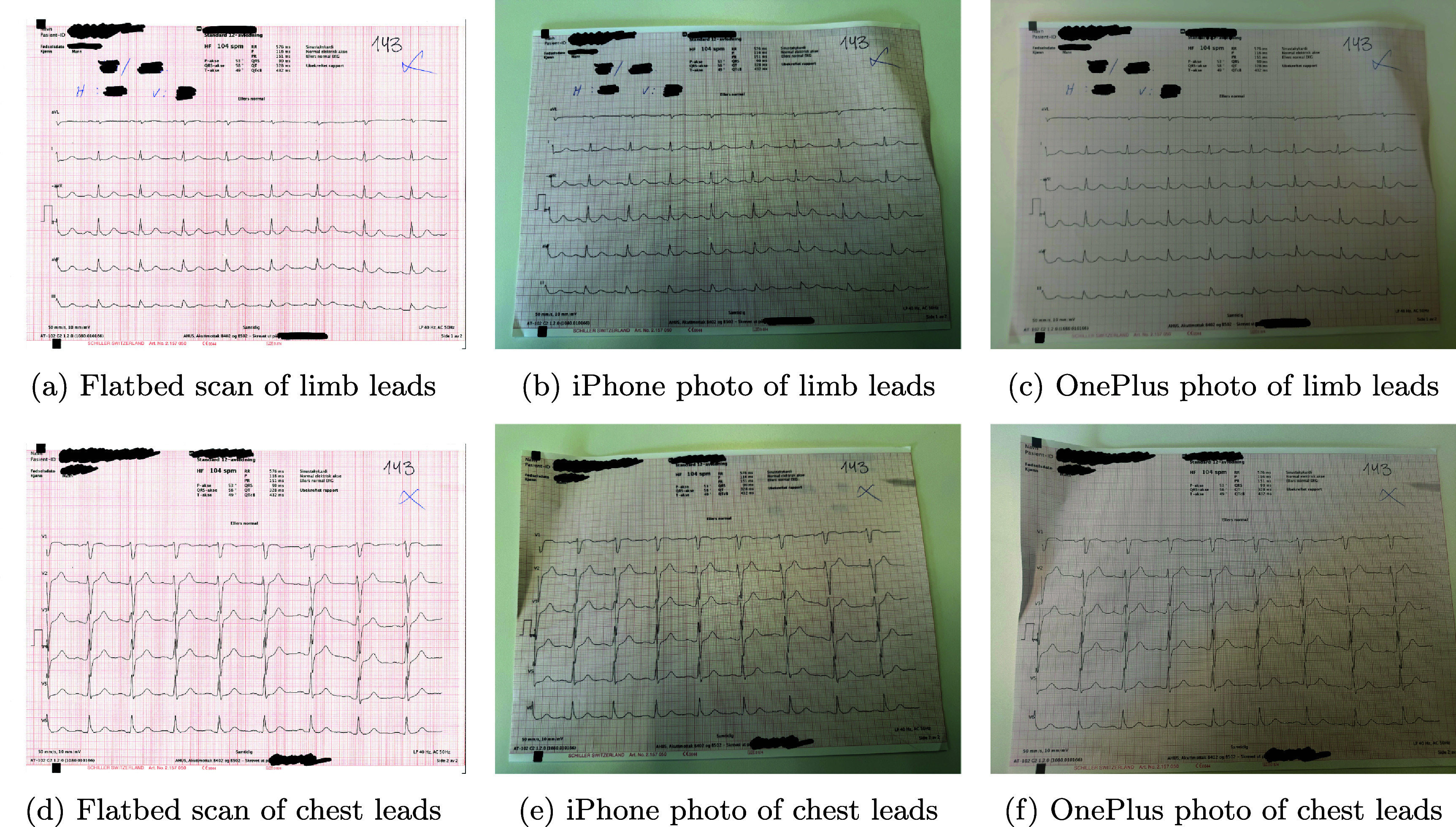
Six variants of an ECG image from the Ahus records after manual redaction of identifiers.

Each ECG page was converted into images using three acquisition modalities: smartphone photography with an iPhone 16 Pro Max ($5712\times4284$ pixels, f/1.78) and a OnePlus Nord CE 2 ($4624\times3468$ pixels, f/1.7), both captured with slight angulation and HDR disabled, and flatbed scanning with a RICOH IM C3500 scanner ($7016\times4964$ pixels, 600 dpi). The smartphone modalities emulate real-world ECG digitization app usage, whereas the flatbed scanner reflects standard hospital workflows and retrospective digitization of legacy ECG archives. Example captures from the three devices are shown in figure 1.

Paper ECGs contained sensitive patient information, including timestamps and direct identifiers, which were manually redacted with black ink before digitization. Indirect identifiers, such as handwritten clinical notes containing weight or height, were also removed. After digitization, a second digital anonymization pass was applied: a heuristic script highlighted all redacted areas in bright green, followed by manual inspection and correction. Finally, all highlighted regions were replaced with solid black pixels to ensure complete removal of sensitive information.

## 5. Dataset preparation

The PTB-XL and Emory time-series data were provided to ECG-Image-Kit to generate initial electronic ECG images. These images were subsequently printed, distorted, and digitized to create multiple electronic and physical representations for ECG image digitization assessment. The resulting dataset variants are described below (see figure 3 for a summary). Examples from the PTB-XL image collection are shown in figure 4. The same workflow was applied to the Emory Healthcare dataset. The Ahus dataset, which contains paired ECG time series and images, was collected and organized separately.

### 5.1. Dataset naming and version control convention

To facilitate comparison among algorithms trained and evaluated on ECG-Image-Database, we adopt a standardized naming convention that supports future dataset expansion. Records are organized under directories named D0 (PTB-XL), D1 (Emory), and D2 (Ahus). Each directory contains subdirectories corresponding to individual records, within which ECG images (e.g. .PNG, .JPG) are provided together with the corresponding time-series data in WFDB format, consisting of .dat signal files and .hea header files. The header files include the sampling frequency, gain required for conversion to physical units, and occasionally additional metadata, all according to the WFDB standard. These data files can be read using standard WFDB-compatible readers.

**Figure 2.**
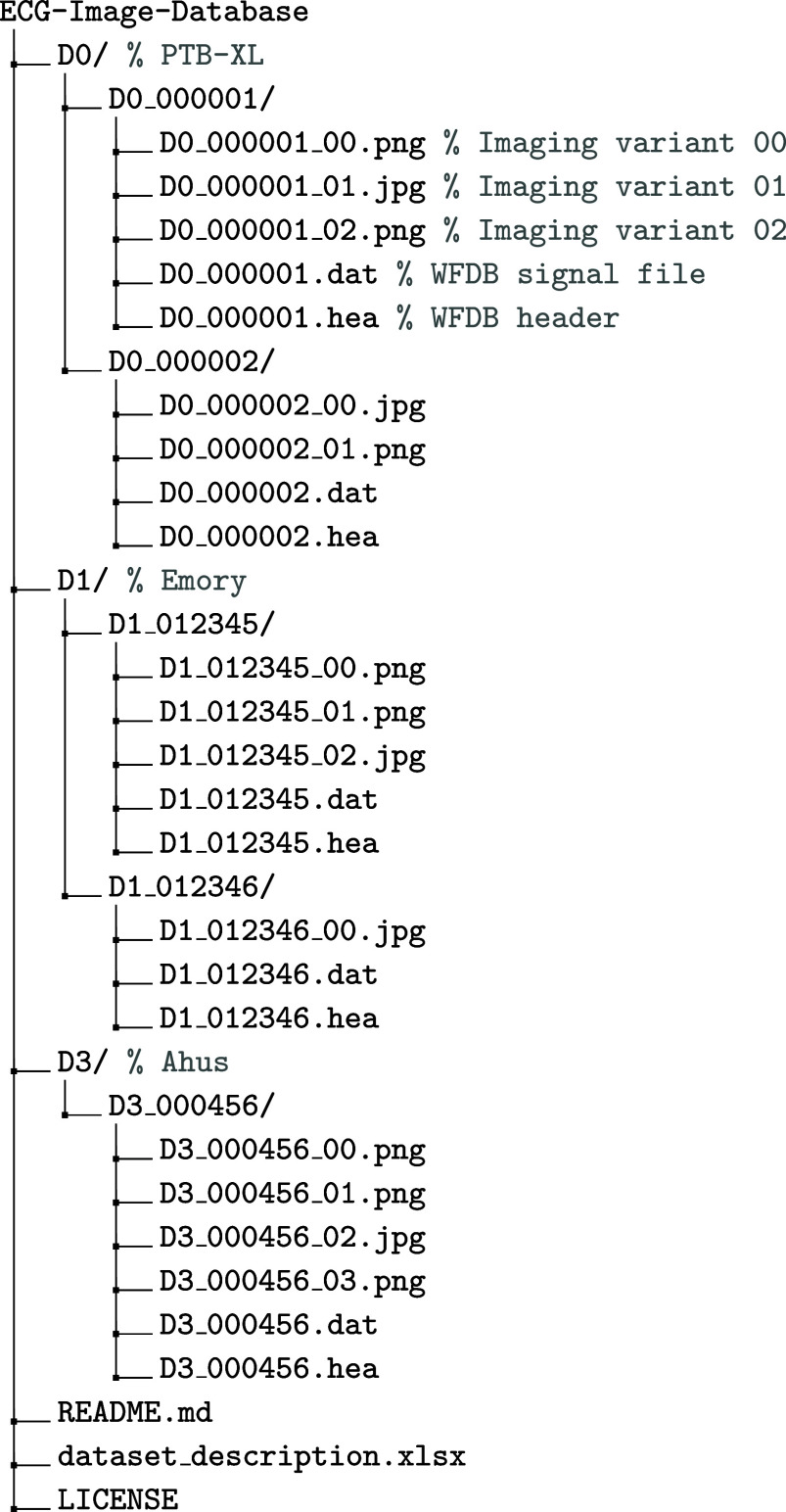
Directory structure and standardized naming convention of ECG-Image-Database. Records are organized by data source under dataset directories (e.g. D0 for PTB-XL, D1 for Emory, D3 for Ahus). Each dataset directory contains record-specific subdirectories holding multiple ECG image representations acquired under different imaging and physical conditions, together with the corresponding time-series data in WFDB format. Image files follow the convention Dn_[RECORD-ID]_[IMAGING-VARIANT-ID], while the associated WFDB signal and header files are named Dn_[RECORD-ID].dat and Dn_[RECORD-ID].hea, respectively.

Each record includes multiple representations of the same ECG, photographed or scanned under different imaging and physical conditions. Image files are uniquely named using the convention Dn_[RECORD-ID]_[IMAGING-VARIANT-ID], where RECORD-ID denotes a unique ECG record identifier and IMAGING-VARIANT-ID enumerates the imaging variants of that record. The corresponding WFDB signal and header files are named Dn_[RECORD-ID].dat and Dn_[RECORD-ID].hea, respectively. For example, record 000 034 from the Emory dataset (D1), organized under the subdirectory D1/D1_00 0034, may contain D1_00 0034_00.jpg, D1_00 0034_01.png, and D1_00 0034_02.png as three imaging variants of the same ECG time series encoded in the WFDB header file D1_00 0034.hea and WFDB signal file D1_00 0034.dat. See figure 2 for the directory structure.

**Figure 3.**
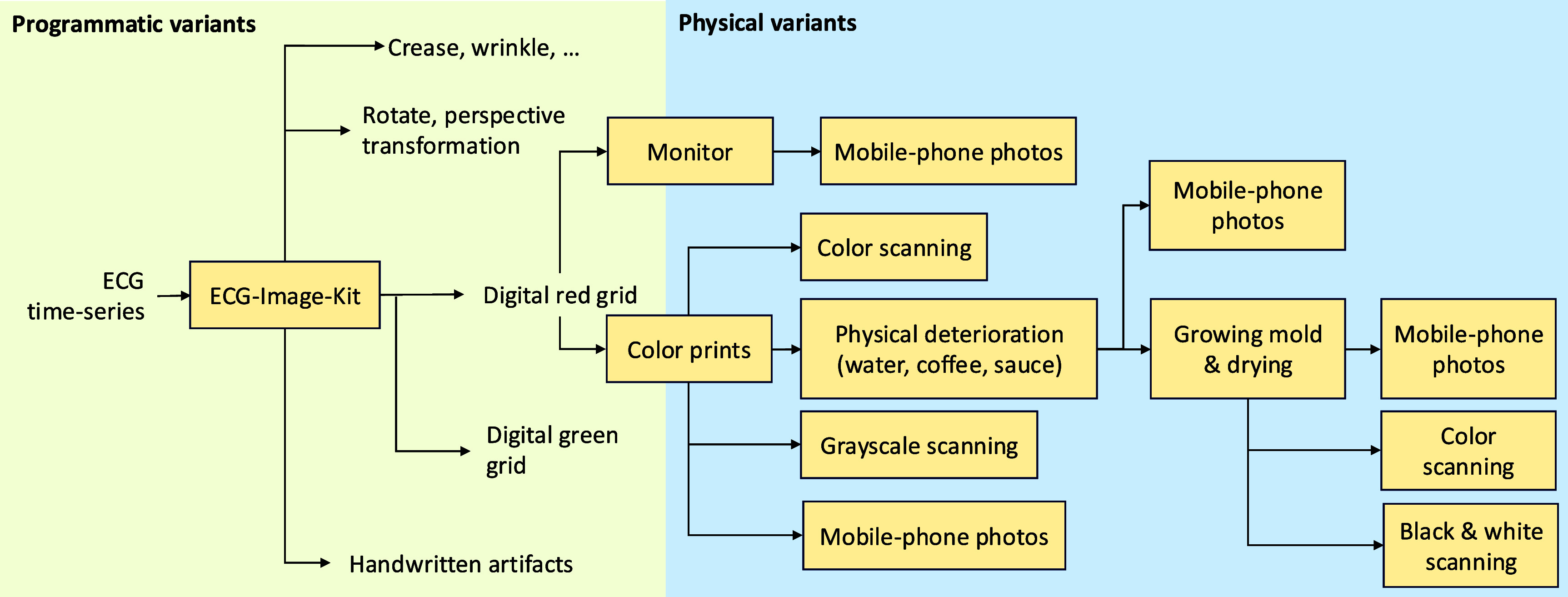
An illustration of ECG-Image-Database creation procedure. The procedure has been repeated for 977 records of the PTB-XL dataset; a similar procedure applies to the 1000 diagnosis-stratified 12-lead ECG records from Emory Healthcare.

**Figure 4.**
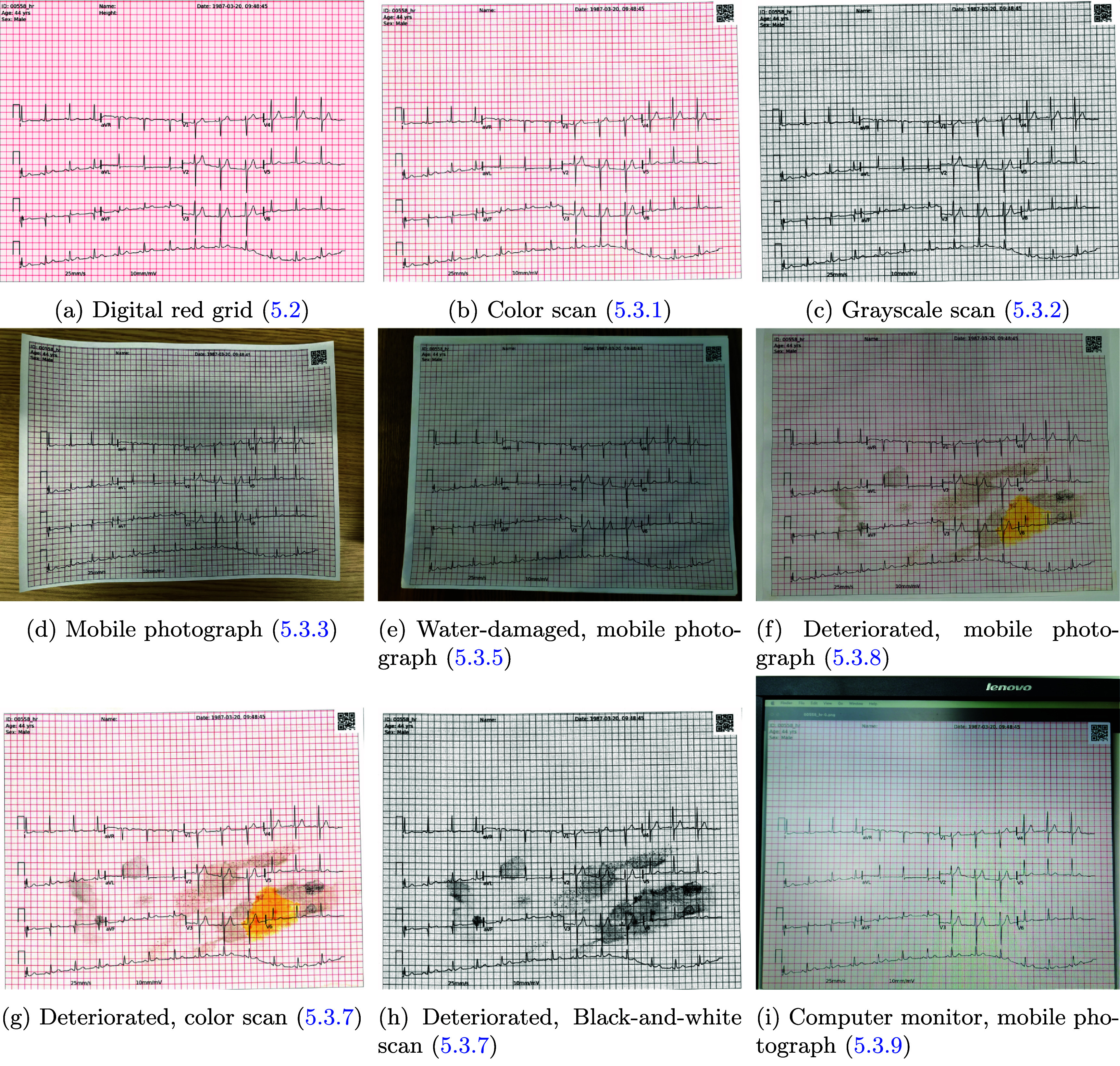
Representative ECG images from ECG-Image-Database derived from the PTB-XL ECG records. Automatically generated QR codes on the top right of each image enable programmatic image-record matching.

The imaging variants available for each data source are summarized in table 1 and described in the ECG-Image-Database README file in Reyna *et al* (2026). In addition, a structured dataset description spreadsheet (dataset_description.xlsx) is provided to document the data sources, imaging variants, and further details regarding the imaging or scanning procedures and devices used for each record. As illustrated in figures 1 and 4, the difficulty of the digitization task varies substantially across records. The spreadsheet can therefore be used to group records by imaging characteristics and to evaluate algorithmic performance across different levels of digitization difficulty.

**Table 1.** Summary of ECG-Image-Database dataset variants derived from ECG time-series from PTB-XL, Akershus University Hospital (Ahus), and Emory Healthcare (EUH). The last column indicates the dataset variant (first column) from which the variant has been derived from (see figure 3).

Dataset variant	Source	Description	Capture modality	Derived from dataset
#1	PTB-XL	ECG images generated from time-series using ECG-Image-Kit, with red ECG grid, saved in PDF.	Electronic (PDF)	—
#2	PTB-XL	ECG images generated from time-series using ECG-Image-Kit, with red ECG grid, saved in PNG.	Electronic (PNG)	—
#3	PTB-XL	Color printouts of synthetic ECG images produced using a laser printer, prior to any physical distortion or degradation.	Hardcopy (paper)	#2
#4	PTB-XL	Clean color scans of printed ECGs acquired using a flatbed scanner, preserving grid color and waveform fidelity.	Scanned image	#3
#5	PTB-XL	Clean grayscale scans of printed ECGs, capturing loss of color information typical of archival scanning workflows.	Scanned image	#3
#6	PTB-XL	Clean prints photographed with mobile phones.	Mobile photo (JPG)	#3
#7	PTB-XL	Original color ECG images photographed from the monitor.	Monitor photo (JPG)	#2
#8	PTB-XL	Primary physical damage applied to printed ECGs including water, coffee, sauce stains, and ironing marks.	Hardcopy (paper)	#3
#9	PTB-XL	Secondary physical damage following primary deterioration, including growing mold and drying effects.	Hardcopy (paper)	#8
#10	PTB-XL	Mobile phone photographs of ECG prints captured post primary deterioration.	Mobile photo (JPG)	#8
#11	PTB-XL	Mobile phone photographs of ECG prints captured post secondary deterioration.	Mobile photo (JPG)	#9
#12	PTB-XL	Color scans of ECG prints post secondary deterioration.	Scanned image (JPG)	#9
#13	PTB-XL	Black and white scans of ECG prints post secondary deterioration.	Scanned image (JPG)	#9
#14	PTB-XL	Synthetic ECG images generated using ECG-Image-Kit with a green grid background, exported in PDF format.	Electronic (PDF)	—
#15	PTB-XL	Synthetic ECG images generated using ECG-Image-Kit with a green grid background, exported as PNG files.	Electronic (PNG)	#2
#16	PTB-XL	Synthetic ECG images generated using ECG-Image-Kit with simulated handwritten artifacts.	Electronic (PNG)	#2
#17	PTB-XL	Synthetic ECG images generated using ECG-Image-Kit with simulated creases and wrinkles.	Electronic (PNG)	#2
#18	PTB-XL	Synthetic ECG images generated using ECG-Image-Kit with rotations and geometric transformations.	Electronic (PNG)	#2
#19	EUH	Synthetic ECG images generated from Emory Healthcare time-series data using ECG-Image-Kit with a red grid, exported in PDF format.	Electronic (PDF)	—
#20	EUH	High-resolution PNG images generated from Emory Healthcare ECG time series using ECG-Image-Kit, intended for training machine learning models.	Electronic (PNG)	—
#21	EUH	Color printouts of synthetic ECG images produced using a laser printer, prior to introduction of physical artifacts.	Hardcopy (paper)	#20
#22	EUH	Clean color scans of printed ECG images acquired using a flatbed scanner.	Scanned image (JPG)	#20
#23	EUH	Clean grayscale scans of printed ECG images, reflecting common clinical and archival digitization practices.	Scanned image (JPG)	#20
#24	EUH	Clean sheets photographed with mobile phone.	Mobile photo (JPG)	#20
#25	EUH	Original color ECG images photographed from the monitor.	Monitor photo (JPG)	#20
#26	Ahus	Flatbed scanner captures of thermal paper ECG printouts collected during routine clinical workflows at Akershus University Hospital.	Scanned image	—
#27	Ahus	Smartphone photographs of thermal paper ECGs captured using an iPhone 16 Pro Max under variable lighting and viewing angles.	Mobile photo	—
#28	Ahus	Smartphone photographs of thermal paper ECGs captured using a OnePlus Nord CE 2 device.	Mobile photo	—

This naming convention will be maintained in future updates to the dataset, whether through new ECG records or new image variants of existing records, and will be documented in the dataset repository.

### 5.2. Generating ECG images from time-series data

For reproducibility, the command-line options used to run ECG-Image-Kit to convert the PTB-XL and Emory datasets into images are detailed in appendix B. Accordingly, the following steps are required to generate images from existing time series records: (i) cloning the required software repositories; (ii) preparing the ECG time-series datasets; (iii) converting the data to WFDB format (Moody *et al* 2022); (iv) generating synthetic ECG images with standardized lead layouts, grid colors, and QR-code identifiers for tracking records after distortion and imaging; and (v) preparing WFDB file headers by removing sensitive metadata. Additional steps include the generation of alternative grid colors and programmatic image distortions, including creases, rotations, and handwritten text, used to create reproducible dataset variants.

### 5.3. Physically distorted variants of the ECG images

We first printed the subset of the Emory and PTB-XL databases, comprising a total of 1977 records, using a LaserJet Pro M501 printer in color mode at a resolution of 600 dpi on US Letter-sized paper. We then introduced physical distortions, including imaging, scanning, photography artifacts, and natural deterioration effects, to the ECG printouts, as detailed below.

#### 5.3.1. Scans of red grid printed in color

The ECG images were scanned on a Brother MFC-L8900CDW color laser all-in-one printer in color mode, in batches of 50–100 images, and saved as multi-page PDF documents. These PDF files were then programmatically split into individual images. The QReader Python package, powered by the YOLOv8 model, was used to detect and extract QR codes containing individual file names from each separated image. These file names were used throughout the dataset creation process to automatically rename the scanned pages to match their original record names. A small number of images with unreadable QR codes were manually matched and resolved.

All images were converted into JPEG format using the Python Python Imaging Library package, with a quality level of 90% (compression level of 10%). Human experts familiar with ECG data performed a visual inspection of the compressed images to ensure that signal readability was maintained. This process reduced the file sizes to approximately 2–3 megabytes per image while preserving an acceptable level of detail for ECG interpretation and waveform extraction.

#### 5.3.2. Grayscale scans of red grid printed in color

The dataset printed in color, including the Emory and PTB-XL datasets, was rescanned in grayscale mode using a Brother MFC-L8900CDW color laser all-in-one printer. The same procedure used for the color scans was followed: the images were saved as multi-page PDF files, which were then split into individual images. An automatic QR code detection system was applied to extract the original waveform filenames from the images. The images were saved in JPEG format with a 90% quality level, using the original filenames obtained through QR code detection.

#### 5.3.3. Photographs of red grid with four different cameras

The red-grid printed ECG images were photographed using four different mobile phones: Samsung Galaxy S20 FE (12-megapixel camera, Emory dataset), iPhone 12 (12-megapixel camera, PTB-XL dataset), iPhone 13 Mini (12-megapixel camera, PTB-XL dataset), and Samsung S10+ (12-megapixel camera, PTB-XL dataset). The photographs were taken in an office setting under ceiling lights. In some images, minor shadows from the cameras, people, or objects were present. All images were renamed using the QR code detector and saved in JPEG format with a 90% quality level.

#### 5.3.4. Soaking and staining distortions

The color-printed ECG images were soaked in water and contaminated with coffee stains and soy sauce. This step was performed to replicate the random deterioration of paper ECGs that can accidentally occur in clinical settings. The effects of water, coffee, and sauce stains varied across images; some were significantly damaged, while others showed moderate or very minor effects. The contaminated printouts were spread out on a table and left to partially air-dry for approximately 12 h under a ceiling fan. Random batches that remained significantly wet were ironed to further dry them, resulting in occasional brownish/tan thermal deterioration or scorching effects. Throughout the experiment, the QR code regions of the ECG images (in the top-right corner of each image) were preserved to facilitate automatic sorting and naming of the corresponding image files.

#### 5.3.5. Photographs of stained and damaged ECG papers

The contaminated printouts were photographed using a Samsung Galaxy S10+ mobile phone under sunlight, partial shade, and ceiling light. Most of the printouts remained moist or partially wet at this stage. The photographs were taken from a distance that allowed the entire paper to fit within the mobile phone camera frame. They were captured from different angles and under various lighting conditions (at night, dawn, and early morning), with varying light levels, resulting in shadows and partial shading in some images. There was no specific order or fixed set of instructions for photographing the ECG records, other than capturing a full image of each ECG. The photographers were given the liberty to take pictures of a quality that would be considered acceptable by people familiar with ECGs.

#### 5.3.6. Growing mold and further drying

The partially moist ECG papers, after being stained with water, coffee, and sauce, were packed into water-resistant envelopes, with approximately 100 images in each. The envelopes were sealed, stacked, and stored in a humid place for six weeks. This caused color stains to expand across pages. Many of the papers developed small, moderate, or significant amounts of mold, further deteriorating the printouts. In some cases, the mold on the coffee and sauce stains interfered with the ECG waveforms, resembling the extreme natural deterioration of paper ECGs over time, as seen in damp or flooded historical hospital archives. The level of deterioration varied across records. Some papers that were relatively dry before packing did not develop any mold or damage.

#### 5.3.7. Scans of moldy red grid; black-and-white and color

The moldy, printed ECG images were scanned using a Brother MFC-L8900CDW scanner. Batches of 10–20 printed images were fed into the scanner to generate multi-page PDFs. Images were first scanned in color mode at 600 dpi resolution and then scanned again in black-and-white mode at 600 dpi resolution. Due to their fragile nature, the moldy, printed ECG images caused frequent scanner jams and were often creased during scanning. Two printed ECGs were destroyed due to paper jamming during the scanning process. These images were reprinted, stained, and rescanned. The resulting PDF files were split into separate image files, renamed using the QR code detector, and saved in JPEG format with a 90% quality level.

#### 5.3.8. Photographs of moldy red grid images

The printed, moldy ECG images were also photographed using two different mobile phones: an iPhone 11 and a Samsung Galaxy S20 FE, both with 12-megapixel cameras.

#### 5.3.9. Photographs of ECG on computer monitors with red grid

Taking pictures of ECG monitors and sharing them with peers for review or with medical trainees is common practice in clinical settings. Technologically, monitors refresh their screens at high rates (e.g. 60 Hz or higher) to provide high-quality images with seamless transitions for the human eye. However, because mobile phones and cameras capture images in very short snapshots, with exposure times comparable to monitor refresh rates and not synchronized with the monitor’s refresh patterns, photos of monitors are often susceptible to unique aliasing and imaging artifacts that affect the color and resolution of the captured images.

To replicate this effect, the Emory and PTB-XL images (original images without physical distortions) were displayed on a Lenovo LT2252PWA 22-inch monitor (1680 $\times$ 1050 resolution) and photographed using a Samsung S22 Ultra mobile phone camera at a resolution of 12 megapixels. To simulate real-life conditions, the lighting, the monitor’s angle relative to the external light source, and the phone’s orientation (landscape or portrait, each with slight variations in angle) were arbitrarily varied by the photographer. The camera distance from the monitor varied between 10 and 15 inches, depending primarily on the orientation. Some blurring was also introduced in some photographs due to hand motion artifacts.

## 6. Discussion

### 6.1. Significance of ECG-Image-Database

With the increasing availability of digital ECGs from modern clinical devices, a vast amount of historical ECG data still exists solely in paper or scanned image form, or as ECGs in electronic health records for which the underlying ECG time series have not been stored or maintained. These legacy records contain vital information about patient health histories, rare cardiovascular events, and demographic-specific data that are irreplaceable for longitudinal studies and population-wide analyses. Without proper digitization and analysis tools, much of this valuable information could be lost due to physical deterioration or the obsolescence of archival systems.

There are limited tools available for digitizing ECG images across different formats. ECG-Image-Database was developed as an effort toward creating standardized reference ECG image datasets paired with the original time-series data. The dataset addresses several key challenges in ECG digitization. By generating a large collection of high-fidelity synthetic ECG images paired with the underlying ECG time series and realistic physical and electronic distortions, the dataset simulates the types of real-world artifacts commonly encountered in clinical practice. These include noise, wrinkles, stains, perspective shifts, and other degradations that often complicate the digitization process. In addition to digitally induced distortions, the inclusion of physically altered images—through soaking, staining, and mold exposure—provides a comprehensive collection of ECG images that mirror those found in real-world clinical and historical archives. This helps ensure that ML and deep learning models trained on this dataset can generalize well to diverse input conditions, an important consideration for widespread adoption in clinical settings and batch digitization of historical archives.

The dataset currently includes three subsets from PTB-XL, Emory Healthcare, and Akershus University Hospital (Ahus). Combined with ECG-Image-Kit’s capability for creating synthetic variants of ECG images, this is a rich resource for developing robust and generalizable ECG digitization software. This may result in models that not only perform digitization (converting image data back into time series suitable for AI/ML applications) but also directly classify conditions based on image data alone, bypassing the need for digitization altogether. Such dual approaches could lead to more efficient tools for ECG analysis by leveraging the complementary strengths of ECGs as both time series and images. As a matter of ongoing debate, clinicians are generally more familiar with the latter holistic approach (examining all ECG leads at a glance), whereas traditional ML pipelines and existing diagnosis tools are primarily based on the former (time series-oriented).

Another important contribution of this dataset is its role in preserving cardiovascular data from non-digital formats. In many low-resource regions, including LMICs, digital ECG infrastructure remains scarce, and healthcare providers often rely on printed ECGs. Even in high-resource settings, paper ECGs are still used in educational settings, clinical decision-making, and peer discussions. ECG-Image-Database can serve as a critical reference for the development of accessible, low-cost digitization tools that can be deployed globally. This opens the door for equitable access to modern cardiovascular diagnostic tools, reducing disparities in health outcomes across regions and populations.

ECG-Image-Database was used as the training, validation, and test dataset for the PhysioNet Challenge 2024: Digitization and Classification of ECG Images (Reyna *et al* 2024), and for a follow-up 2025–2026 Kaggle challenge (Reyna *et al* 2025). These challenges encouraged the development of open-source methods combining conventional signal processing, computer vision, and deep learning to extract time-series signals from ECG images with varying levels of degradation. In the Kaggle challenge, top-performing methods achieved reconstruction performance exceeding 22 dB SNR, corresponding to waveform differences that are often visually negligible to human experts. Follow-up analyses of fiducial points and ECG biomarkers extracted from the digitized signals further showed statistically similar performance compared with the original time-series data, including for PT interval, QT interval, QRS duration, heart rate, and ST-segment measurements. These results suggest that ECG digitization approaches built on ECG-Image-Database may already be sufficient for many clinically relevant ECG analysis tasks under moderate imaging conditions, although substantial room remains for improving robustness under extreme distortions and artifacts. Our intent is to maintain ECG-Image-Database (Reyna *et al* 2026) as a living repository, frequently updated with additional real-world ECG images and time-series data collected under more challenging conditions to support ongoing advances in ECG digitization, image-based analysis, and benchmarking.

### 6.2. Limitations

Despite these advances, several challenges remain. One important limitation is that the majority of ECG-Image-Database records consist of controlled physical and electronic degradations of ECG printouts and partially synthetic (programmatically generated) distortions. Although these distortions were designed to emulate realistic imaging conditions, and the Ahus subset provides real-world paired ECG images acquired during routine clinical workflows, the current study does not include a systematic quantitative comparison between synthetic-derived and real-world ECG images using existing ECG digitization algorithms or software. As a result, the extent to which synthetic images fully capture the variability and challenges of real-world ECG digitization remains an open question.

A related limitation is the current lack of standardized and publicly available ECG digitization software suitable for objective benchmarking. Although several ECG digitization methods have been reported in the literature, we were unable to identify publicly available codebases that generalized reliably to the dataset collected in this work. To the best of our knowledge, the only currently available software with robust performance on raster ECG images is a commercial product, PMcardio (Demolder *et al* 2025), which is not available as an open-source codebase. This limitation was indeed a major motivation for organizing the PhysioNet Challenge 2024 and 2025–2026 Kaggle challenge using ECG-Image-Database, which have already resulted in numerous publicly released ECG digitization algorithms. However, because these methods were themselves developed and evaluated using ECG-Image-Database, it would be scientifically circular to use them to validate the dataset within the present work.

Another challenge is dealing with low-quality images, especially those captured under suboptimal conditions such as poor lighting, incorrect angles, or damaged physical ECGs. While ECG-Image-Database provides a range of such examples, real-world variability is difficult to fully capture in a controlled setting.

The most common single-page 10 s ECG printouts often interleave the leads, for example by printing 2.5 s segments from each of the twelve leads, plus a full 10 s rhythm strip from one to three leads. Thus, most segments of a 10 s 12-lead recording are dropped during printing, and even perfect digitization cannot recover the missing segments. We recently used the natural redundancies between ECG leads and morphology-aware lead imputation to recover parts of this missing information (Hassannia *et al* 2026); however, the problem remains an intrinsic limitation of ECG image digitization.

ECG images with PHI also remain an important challenge. While ECG-Image-Kit provides tools for generating printed and handwritten-style text on generated ECG images, the dataset does not currently host substantial examples of these cases, which could be used to train ML algorithms to remove PHI and/or evaluate robustness to PHI leakage or adversarial attacks. The Ahus subset includes limited examples with handwritten text, which should be expanded and diversified in future updates.

Another aspect requiring further exploration is performance metrics for ECG image digitization. The PhysioNet Challenge 2024 and the 2025–2026 Kaggle challenge employed a modified SNR metric, insensitive to horizontal and vertical shifts, to measure the quality of ECG image reconstruction by participating teams, which was feasible because ECG-Image-Database provides the ground-truth time-series data. However, for real-world ECG images where ground-truth data are unavailable, calculating SNR is infeasible. Moreover, SNR is not necessarily the best metric for assessing the fidelity of a digitization algorithm in preserving ECG details that are significant for ECG-based diagnosis. While it captures major discrepancies—such as distortion in or loss of the QRS complex or T-wave—it might not fully reflect the clinical significance of more subtle and low-power ECG details, such as the Q-wave onset/T-offset or the ST segment, which are crucial for accurate diagnosis.

Finally, although the dataset includes a broad range of artifacts and acquisition conditions, it cannot fully represent the variability of real-world ECG workflows across institutions, devices, paper formats, scanners, and mobile capture settings. External validation on independently collected ECG image datasets therefore remains important for evaluating generalizability.

### 6.3. Future work

In future research, ECG-specific signal quality metrics (e.g. weighted SNR measures) could be developed to better quantify preservation of diagnostically important waveform features. More clinically meaningful evaluation frameworks may help assess whether digitized ECGs preserve the information required for biomarker extraction and accurate diagnosis, rather than simply maximizing waveform similarity. In many applications, preserving the diagnostic interpretation of the ECG may ultimately be more important than maximizing conventional reconstruction metrics such as SNR.

We will conduct follow-up studies to benchmark and compare ECG digitization algorithms developed through the PhysioNet Challenge 2024 and 2025–2026 Kaggle challenge, including evaluation of their strengths, limitations, biomarker recovery performance, and generalization across synthetic and real-world ECG subsets. These analyses will also compare open-source approaches against commercial solutions where feasible.

Future work should also expand the diversity of ECG-Image-Database through additional ECG devices, healthcare systems, acquisition conditions, and ECG formats. Expanding the dataset to include more examples with handwritten annotations, PHI, and severe real-world degradation would further improve the robustness of future ECG digitization models. Because real-world ECG images are highly heterogeneous, ECG-Image-Database is intended to remain a continuously evolving and version-controlled resource that can grow as additional paired ECG image and time-series datasets become available.

In addition, future studies may increasingly explore direct image-based ECG interpretation methods that bypass explicit waveform reconstruction altogether. Such approaches could complement traditional signal-based pipelines and further leverage the complementary strengths of ECGs as both time series and images.

Finally, continued community benchmarking efforts and open evaluation protocols will remain important for advancing reproducible and clinically relevant ECG digitization research. Continuous updates to ECG-Image-Database and ECG-Image-Kit are expected to support the development of more robust and generalizable ECG image analysis systems.

## 7. Conclusion

ECG-Image-Database offers a standard resource for developing machine- and deep-learning models to digitize and classify ECG images by reproducing real-world challenges such as image distortions, noise, and environmental artifacts. By replicating the conditions under which ECGs are stored and scanned, the dataset helps ensure that models trained on it can effectively handle real-world complexities and preserve valuable diagnostic information from paper-based ECG records.

ECG-Image-Database will remain a living repository, allowing updates that support both clinical and low-resource applications in cardiovascular diagnostics. Future expansions, including additional image sources and further environmental variations, will enhance the dataset’s utility for training models that generalize across diverse conditions. The database is structured and organized to facilitate expansion.

## Figures and Tables

**Figure 5.**
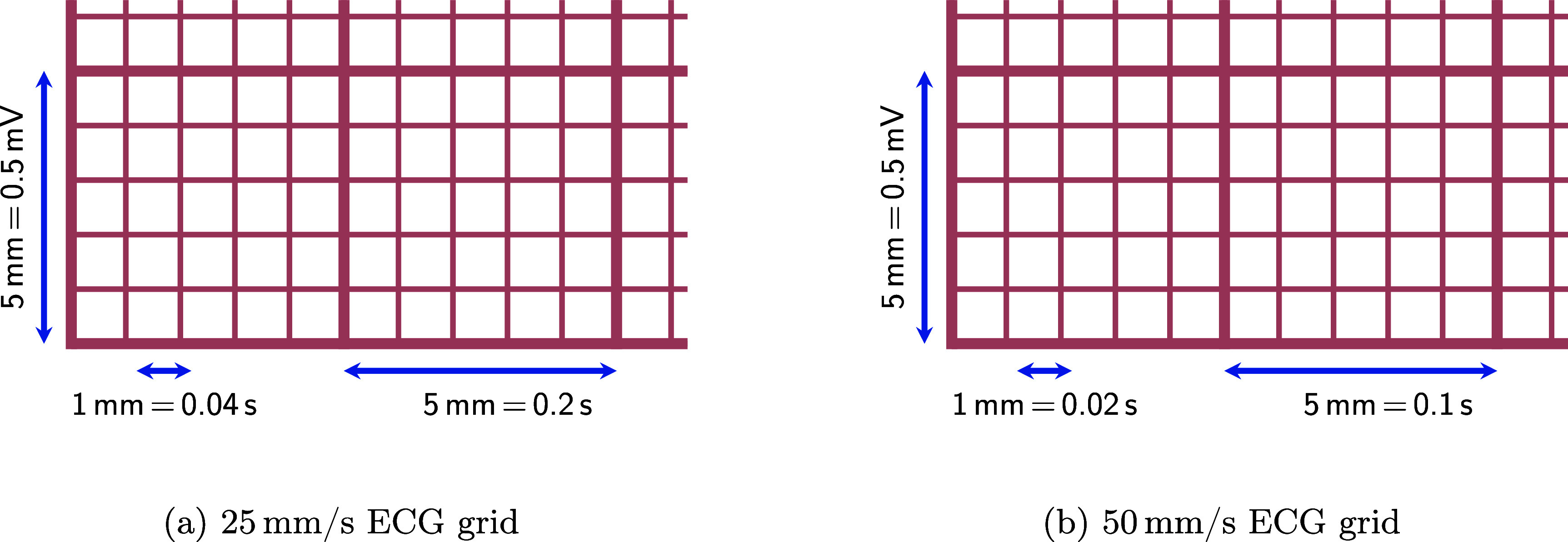
Standard grid lines printed on both hardcopy printouts and digital ECGs and used by clinicians (and algorithms) as references for measuring time and amplitude units of an ECG. The voltage resolution is the same across all paper ECGs in the dataset. In ECG-Image-Database, the Emory and PTB-XL records include images with 25 mm s$ ^{-1}$ time resolution, while the Ahus records includes images with 50 mm s$ ^{-1}$ time resolution.

## Data Availability

The data that support the findings of this study are openly available at the following URL/DOI: https://www.kaggle.com/datasets/physionet/ecg-image-database/ (Reyna *et al* 2026). Data will be available from 01 August 2026.

## Appendix A Notes on ECG image vs time-series resolutions

### A.1. ECG as a time series

The ECG recorded by standard body-surface leads typically has an amplitude of several millivolts, and its spectral content ranges from approximately 0.05 Hz to around 150 Hz. When transformed into a digital signal, it first passes through an anti-aliasing low-pass filter in the analog domain before being sampled at a given sampling frequency $f_\mathrm s$. According to the Nyquist theorem, the cutoff frequency of the anti-aliasing filter should be below $f_\mathrm s/2$. ECG systems with lower resolution, such as older single-lead machines, Holter monitors, and wearable devices, may have sampling frequencies as low as 100 Hz. Modern high-quality clinical monitors often sample at higher frequencies, such as 1000 Hz or higher.

The amplitude resolution of the digital signal depends on the number of bits of the analog-to-digital converter (ADC), denoted by $N$. In older ECG devices, $N$ was as low as 8 bits, resulting in a maximum resolution of 256 quantization levels, assuming that the ECG was amplified to span the ADC’s full dynamic range. In modern ECG devices, $N$ can be up to 24 bits, resulting in significantly better amplitude resolution and dynamic range, defined as the ratio of the highest to the lowest detectable amplitude components of the signal. Importantly, due to electronic and thermal noise, and depending on the quality of the analog front-end circuitry, the effective number of bits (ENOB) is, in practice, lower than the nominal ADC bit number $N$. For instance, a 16-bit ADC may yield between 12.5 and 14 ENOBs in practice, often several bits below the nominal number of bits $N$. With an $N$-bit ADC digitizing the input voltage range from $V_{\min}$ to $V_{\max}$, the voltage resolution after digitization is:

\begin{equation*} \delta v = \frac{V_{\max} - V_{\min}}{2^N}\end{equation*}

For example, if the input span of the ADC is $\Delta V = V_{\max}-V_{\min} = 5\,\mathrm{mV}$, an 8-bit ADC yields a voltage resolution of $\delta v = 19.5\,\mu\mathrm{V}$, while a 12-bit ADC yields $\delta v = 1.22\,\mu\mathrm{V}$, improving the voltage resolution by a factor of 16.

### A.2. Printed ECG

In clinical applications, ECGs are printed on standard ECG paper featuring fine and coarse grid lines. Following long-established conventions originating from analog strip-chart ECG recorders, the horizontal paper speed is typically 25 mm s$ ^{-1}$ or 50 mm s$ ^{-1}$, and the vertical scaling is most commonly 10 mm per 1 mV. Under these conventions, the fine grid measures 1 mm by 1 mm, corresponding to 0.1 mV in amplitude and 40 ms or 20 ms in time for paper speeds of 25 mm s$ ^{-1}$ and 50 mm s$ ^{-1}$, respectively. The coarse grid measures 5 mm by 5 mm, corresponding to 0.5 mV in amplitude and 200 ms or 100 ms in time for 25 mm s$ ^{-1}$ and 50 mm s$ ^{-1}$ paper speeds, respectively, as shown in figure 5.

In modern ECG devices, despite the data being collected digitally and stored on computers or other digital platforms, the same convention is used. ECGs are visualized against the same background grids, whether displayed on a computer screen or printed as a PDF or image file. The number and format of the leads, along with the ECG grid color, vary depending on the ECG acquisition technology and device manufacturer. In the U.S., the most common clinical ECGs (by GE or Philips) are 12-lead ECGs, featuring approximately 2.5 s segments of the 12 leads arranged in a 3-row by 4-column grid. Additionally, one to three leads (typically leads II, V1, V2, or V5) are displayed as longer 10 s strips at the bottom. In Europe, the aforementioned format is sometimes used in emergency settings. A more common layout is the 6-by-2 format, in which the limb leads are displayed first, followed by the precordial leads V1–V6 in order, either on the same or separate pages. Many ECG devices allow users to configure the layout through software settings.

### A.3. Scanned ECG images

Printing an analog or digital ECG on paper and then rescanning it as an image involves implicit or explicit interpolation and resampling of the original ECG. When performed by an analog machine or a standard printer, this process involves digital-to-analog circuitry to convert the discrete-time samples into a continuous waveform, printed as a continuous curve on paper. Once an ECG is printed, the original sampling frequency $f_\mathrm s$ of the digital time series and the number of quantization bits $N$ become irrelevant, as the signal has been transformed back into the continuous time and amplitude domain. When an ECG paper is scanned or photographed as an image, it is essentially quantized and resampled again, this time as a two-dimensional image. Assuming the image is scanned at a resolution of $D$ dots per inch (DPI), each 1-inch by 1-inch square of the printed ECG is quantized into a $D \times D$ array of pixels, with each pixel stored in $B$ bits. This is different from $N$, the number of ECG acquisition ADC bits. If scanned as a color image, there will be three such arrays, corresponding to the red, green, and blue color channels. Modern images typically use $B = 8$, resulting in 24 bits, or 3 bytes, per pixel. Therefore, for example, scanning a letter-size paper of 11 inches by 8.5 inches at 72 DPI would require $8.5 \times 11 \times (72 \times 72) \times 3$ bytes = 1454 112 bytes, or 1.39 MB, as an uncompressed bitmap file, excluding any metadata or file headers. In practice, fewer bits, corresponding to lower color depth, and/or lossy or lossless image compression can reduce the storage needed to save such an image with minimal or no loss of information, although scanning and other artifacts may impede compression. Imaging at a distance, such as mobile phone photography, reduces the effective DPI allocated to the ECG paper because some of the resolution is spent on the background.

When a standard ECG, printed on A4 or letter-size paper, is scanned at full image size without cropping or excess borders, each 1 inch (25.4 mm) horizontally and vertically maps to $D$ pixels. In other words, each coarse square of the ECG (0.5 mV in amplitude and 200 ms or 100 ms in time) maps to a square of $\left(\frac{5 \times D}{25.4}\right)\times\left(\frac{5 \times D}{25.4}\right)$ pixels. This means that the amplitude resolution of the scanned ECG *per pixel* is:

\begin{equation*} \mathrm dv = \frac{2.54}{D} \textrm{mV}\end{equation*}

and the temporal resolution *per pixel* is $\mathrm dt = \frac{1.016}{D}$ seconds for 25 mm s$ ^{-1}$ paper and $\mathrm dt = \frac{0.508}{D}$ seconds for 50 mm s$ ^{-1}$ paper. Equivalently, the sampling frequency (in Hertz) of the ECG as an image is:

\begin{equation*} f_\mathrm s^{^{\prime}} = \frac{D}{1.016}\quad(25 \text{mm s}^{-1} \mathrm{paper}), \quad f_\mathrm s^{^{\prime}} = \frac{D}{0.508}\quad(50 \text{mm s}^{-1} \mathrm{paper}).\end{equation*}

As shown, the effective sampling frequency $f_\mathrm s^{^{\prime}}$ of the scanned ECG is independent of the original digital signal’s sampling frequency $f_\mathrm s$. However, since the original signal’s frequency range was limited to $f_\mathrm s/2$ by the analog front-end anti-aliasing filter, increasing $D$, and therefore $f_\mathrm s^{^{\prime}}$, will yield smoother waveforms but will not add information beyond $f_\mathrm s/2$, the Nyquist rate of the original digital ECG time series.

From (3), it is evident that typical image resolutions, such as 72 or 96 DPI, which are common in image analysis applications, are quite low for ECG scanning and digitization applications; for 25 mm s$ ^{-1}$ papers, they provide sampling frequencies of only 70.9 Hz and 94.5 Hz, respectively. An ECG should be captured at least at 125 Hz, although 250 Hz, or even 500 Hz, is preferable. Based on this analysis, a resolution of at least 150 DPI or higher, without lossy compression, is recommended for ECG scanning and digitization purposes.

Importantly, the calculation of ECG grid size from image DPI and paper size is accurate only when using a standard full-paper-size scanner. For ECG images captured by cameras, smartphones, screenshots, or through cropping and resizing, the equivalence of 1 inch on the actual paper to the captured image DPI may not hold. Consequently, ECG digitization algorithms should estimate the correct grid size using algorithms that detect and analyze the ECG grid directly from the ECG image. Several functions for this purpose are provided in ECG-Image-Kit (Deepanshi *et al* 2024). Further details and examples can be found in Shivashankara *et al* (2024).

## Appendix B ECG-Image-Kit commands used to generate ECG images from time series

For reproducibility, we review the ECG-Image-Kit (Deepanshi *et al* 2024, Shivashankara *et al* 2024) commands used to convert the PTB-XL and Emory datasets into images for the 2024 PhysioNet Challenge. The 2025–2026 Kaggle challenge used a specific format commonly used in Kaggle challenges; read more in Reyna *et al* (2025). We only review the former commands.

### B.1. Images with red grid

In order to convert an ECG time series record to an ECG image, the following stages are required:

1. *Step 0: Clone the required repositories.* Clone ECG-Image-Kit: https://github.com/alphanumericslab/ecg-image-kit and the PhysioNet Challenge 2024 codebase: https://github.com/physionetchallenges/python-example-2024.git,
2. *Step 1: Downloading the time-series datasets.* In our open-access dataset, the Emory Healthcare time-series data is provided in WFDB format, with annotations included in the WFDB header files. The PTB-XL and PTB-XL+ datasets are not hosted in our repository in time-series format, but they are publicly available on PhysioNet (Goldberger *et al* 2000, Wagner *et al* 2020, 2022, Strodthoff *et al* 2023). PhysioNet provides both interactive and programmatic download options. If downloaded in compressed formats, the data should be unzipped for subsequent processing.
3. *Step 2: Convert the data to WFDB (WaveForm DataBase format).* This can be done using the following script from the Reyna (2024) for the PTB-XL and PTB-XL+ datasets. The data argument is a folder with records from the PTB-XL dataset, and the other files are metadata from the PTB-XL or PTB-XL+ datasets.
  python prepare_ptbxl_data.py \
          -i data \
          -pd ptbxl_database.csv \
          -pm scp_statements.csv \
          -sd 12sl_statements.csv \
          -sm 12slv23ToSNOMED.csv \
          -o data
4. *Step 3: Generate synthetic ECG images.* Next, we generate synthetic ECG images from the dataset using the following command from ECG-Image-Kit:
  python gen_ecg_images_from_data_batch.py \
          -i data \
          -o data \
          –add_qr_code \
          –print_header \
          –mask_unplotted_samples \
          –store_config 2
  The ECG-Image-Kit command line options selected above and the values for the parameters with default values are listed in table 2. More specifically, gen_ecg_images_from_data_batch.py uses the default parameters and flags to generate ECG images with red grids in the background. The –add_qr_code flag generates QR codes containing the file names in the top-right corner of the generated ECG images. QR codes, or Quick Response codes, are two-dimensional pictographic codes that provide faster/programmatic readability and greater storage capacity than traditional barcodes. In our application, the QR codes facilitate the automatic batch scanning and renaming of the scanned/photographed images, ensuring that the reference time series and the corresponding ECG images match one another for building machine learning models. For this step, we used the Python module named qrcode, which utilizes the Python Imaging Library (PIL) to create QR codes. This module offers both simple and advanced usage options, allowing users to generate QR codes with various levels of customization. The qrcode module allows users to control parameters such as version (size), error correction level, box size, and border thickness, enabling the creation of QR codes tailored to specific requirements. Using the qrcode library, we encode the signal filename used to generate the corresponding synthetic image.
5. *Step 4: Add image file locations to WFDB header files.* Add the file locations and other relevant information for the synthetic ECG images to the WFDB header files. This can be accomplished with the following command:
  python prepare_image_data.py \
          -i data \
          -o data \
6. *Step 5: Prepare data for training/inference (optional).* To prepare a version of the data for inference (for instance to train models for ECG image digitization or classification), the user will need to remove the time-series waveforms, certain information about the waveforms (like the diagnoses and/or the demographics). This version of the data can be used for the inference step.
  python gen_ecg_images_from_data_batch.py \
          -i data \
          -o hidden_data \
          –add_qr_code \
          –print_header \
          –mask_unplotted_samples
  python prepare_image_data.py \
          -i hidden_data \
          -o hidden_data
  python remove_hidden_data.py \
          -i hidden_data \
          -o hidden_data \
          –include_images

**Table 2.** Default parameters of gen_ecg_images_from_data_batch.py used to generate ECG images with red grids as described in section B.1.

Parameter	Value	Description
resolution	200	Image resolution
num_columns	4	Number of columns for leads in the ECG image
full_mode	II	Leads to add as long strips at the bottom of the ECG image

### B.2. Images with green grid:

Some ECG device manufacturers use paper with non-red grid colors. ECG-Image-Kit supports arbitrary grid colors through its command line parameters. We used the following command (instead of the command in Step 6 above) to generate green ECG grid paper, which is common in some ECG machines:

python gen_ecg_images_from_data_batch.py \

-i data \

-o hidden_data \

–add_qr_code \

–print_header \

–mask_unplotted_samples \

–standard_grid_color 4 where 4 is the color code for green.

### B.3. Programmatic distorted variants of the ECG images

ECG-Image-Kit can be configured to generate realistic ECG image distortions that resemble real-world paper distortions. ECG-Image-Database hosts several variants of the PTB-XL and Emory datasets with these programmatically generated distortions, which are available for reference comparisons in ECG digitization research. Due to the stochastic feature of the toolbox in generating random distortions, similar commands can be used to generate much larger datasets with similar realistic distortions for training data-demanding deep models.

#### B.3.1. Creases

Creases are simulated by adding evenly-spaced lines to mimic paper fold creases. Gaussian blurring, a common image augmentation technique, is applied to these lines to introduce smoothing effects. The blurring enhances realism by creating a shadow-like effect in the creases, common in scanned images or real paper-ECG. Wrinkles, on the other hand, are treated as textures, which can be generated using texture synthesis methods like image quilting. To add the wrinkles and creases to the generated images, we use the –wrinkles flag and set crease angle (-ca) to the desired angle (45 degrees in the example below):

python gen_ecg_images_from_data_batch.py \

-i data \

-o data \

–wrinkles \

-ca 45 \

-se 10 \

–random_grid_color \

–add_qr_code

#### B.3.2. Rotations

To add rotations to the generated images, we set the augment attribute and set rotation flag (-rot) to the desired angles (30 degrees in the example below), the cropping percentage (-c) value (0.1 in the example below) and select –deterministic_rot and –deterministic_noise flags. The deterministic flags ensure a deterministic behavior with no randomness, guaranteeing reproducibility of the process. The full command line is as follows:

python gen_ecg_images_from_data_batch.py \

-i data \

-o hidden_data \

–augment \

-rot 30 \

-c 0.1 \

–deterministic_rot \

–deterministic_noise \

-se 10

#### B.3.3. Handwritten text

To add handwritten text to the images, we set the hw_text flag and set the number of words to add (n) in handwritten style (4 in the example below), the horizontal offset of the added text (x_offset) and vertical offset (y_offset) in pixels (30 and 20 pixels, respectively, in the example below).

python gen_ecg_images_from_data_batch.py \

-i ptb-xl/records500/00 000 \

-o ptb-xl/records500_hidden/00 000 \

–hw_text -n 4 \

–x_offset 30 –y_offset 20 \

-se 10 –random_grid_color \

–add_qr_code
